# Report on the Physical Welfare of Women and Children

**Published:** 1917-06-30

**Authors:** 


### THE PAST AND THE OUTLOOK IN MIDWIFERY.

### Report on the Physical Welfare of Women and Children.

By THE CARNEGIE UNITED KINGDOM TRUST.

The two solid volumes of the Carnegie Trust Report on the Welfare of Mothers and Children will probably—unfortunately—be chiefly read by experts. Even to those who should be well-informed, however, much of the contents, especially of Dr. Janet Campbell's admirable second volume, and of the condensed reports of local medical officers, will be new, and should be highly instructive. At a time when betterment of every detail in the environment and development of our child population has been anxiously studied, and efforts for the mothers have not been wanting, attention is at last called to the position of those women whom Peter Chamberlen in the seventeenth century called "the uncontrolled arbiters of life and death." Dr. Campbell's brief but deeply interesting historical sketch shows that while in France the *sage femme*, as her very name showed, has always held an honoured position, in England the midwife's status has been less satisfactory. Superstition and ignorance have often exposed mother and babe to greater peril from women claiming to be skilled than if left to the care of a mere friendly neighbour, who would wait upon the process of Nature. Child-birth has been viewed as a natural process and as a form of disease, as a matter for women or a crisis for the doctor, a process of Nature or an occasion for science.

The contest between midwives and male rivals constantly recurred. Abroad, Italian women of position demanded the attendance of a doctor. In 1580 Duke Ludwig of Würtemberg ordained that herdsmen and shepherds should not attend women, and the Princess Palatine, daughter of James I., fetched one Margaret Mercer from England to Heidelberg for her own need. English women frequently objected to the *accoucheur*; Willughby, on one occasion, disguised himself as a woman to help his daughter with a critical case. But the idea of organised training only occurred occasionally to some specially intelligent person. About 1633 Peter Chamberlen taught some few women. He belonged to the family of obstetric surgeons who had rediscovered the use of the forceps, lost since Avicenna's tenth-century practice, and later at Amsterdam, where his son, Hugh, worked in Van Deventer's school, his "secret" was regularly sold by the examiners to medical students as a condition of a diploma. James II. "considered" the plans of a College for Women proposed by Elizabeth Cellier. In the eighteenth century Richard Manningham received poor mothers in his own house in order to train midwives, and a Mrs. Stephen published a text-book, "The Domestic Midwife." Mrs. Nihell was famed as having trained in Paris. In France Mme. Le Boursier du Coudray worked at this time as travelling teacher, with a mannequin approved by the Académie de Chirurgerie. In 1745 the Dublin Rotunda began its excellent work, and the foundation of Queen Charlotte in 1752 and Westminster in 1765 trained a few women. But in 1816 Dr. Merriman could claim that the reduction of infant deaths from 1 in 19 to 1 in 30 was due to the wider employment of men. It must be remembered that midwives often thought it impiety to revive a stillborn infant. Also their practice of laying-out a patient dead from puerperal fever and then at once taking another case must have caused much loss of life. No doubt many of their practices were often very risky, sometimes worse. But when Miss Nightingale's passion for statistics urged Dr. Farr's inquiry of 1869, it appeared that they attended a vast proportion of cases and that no organised training existed. Her further inquiry in 1871 elicited grim facts as to the mortality in institutions, shown to be fifteen times heavier, however, in Paris. The permissive examination and optional diploma of 1870 brought no organisation into being, and, while evidence taken by the Select Committee of 1902 showed the danger of midwives' ignorance of antiseptic precautions and infant management, the formation of the Board, as we know, was delayed till 1902. The anxiety then felt as to the possible results of creating a new class of medical practitioner has acquired a new importance in our own day. The chief fault to be found with the Russian *feldsher*, semi-medically trained for maternity, vaccination, etc., seems to be that the 24,000 men and 6,000 women are too few for the vast country districts of the Empire.

Our outlook is now upon a highly complicated problem. Its details are admirably stated in the Report, and valuable facts with some noteworthy suggestions emerge from the local medical officers' statements. Thus the suggestion that the "handy woman" who must continue to be often preferred to a midwife or doctor by the very poor on account of her usefulness in the home should be registered and watched over, and to some extent instructed, deserves consideration. When we realise that of 40,513 names on the present Roll, 9,529 are accepted as untrained, it is evident that vigorous effort for increase of opportunity of learning, and for the best teaching, is needed, as well as effort to bring home to the uneducated the importance of proper care for mother and child at the critical time. Equally evident is the need for prenatal care in certain cases; the foundation of observation and rest homes, as at Liverpool, marks progress in this respect. Another step is marked by experiment in residential *crèches* for children during the mother's absence for special treatment. The increase of students taking the C.M.B. diploma in order to obtain posts as health visitors, etc., is a rather disquieting fact. Certainly the position of the practising midwife is very difficult; it is an added grievance if young University women become local superintendents with some such qualification. It is, or may soon become, another grief that the diploma is alike for the village woman who barely scrapes through an examination and for the most highly trained and experienced. Meantime the roused sense of her value should bring improvement—*must* do so if a good class of women is to be attracted—to the status and financial position of the professed midwife. At present neither can be thought commensurate with her heavy responsibilities. She may have to wait long for a good practice—at Eastbourne three qualified women shared eleven cases in the year, unqualified old women and friendly neighbours holding the field. Poor cases pay little, there may be bad debts, the cost of drugs and appliances, perhaps a doctor's fee, the need to employ labour in her own home, cases lost through contact with infection—one midwife is recorded to have lost seven through contact with a diphtheritic child—her own maintenance between cases, all are heavy charges.

The most cheering point about the Report as a whole is, however, the indication it gives of public interest throughout the country being so aroused to the questions of mother and child welfare that they are not likely again to be shelved, however complicated and tedious may be their real solution.

